# A new player in the beneficial effects of exercise on the aged brain

**DOI:** 10.1038/s41392-020-00305-5

**Published:** 2020-09-03

**Authors:** M. Llorens-Martín

**Affiliations:** 1 Department of Molecular Neuropathology, Centro de Biología Molecular “Severo Ochoa”, CBMSO, CSIC-UAM, Madrid, Spain; 2 Center for Networked Biomedical Research on Neurodegenerative Diseases (CIBERNED), Madrid, Spain; 3 Department of Molecular Biology, Faculty of Sciences, Universidad Autónoma de Madrid, Madrid, Spain

**Keywords:** Regeneration and repair in the nervous system, Molecular neuroscience

In a recent article in *Science*, Horowitz et al. identify glycosylphosphatidylinositol (GPI)-degrading enzyme Gpld1 as the most critical player in the blood that can transfer the effects of exercise on adult neurogenesis and cognition to sedentary aged mice. They also demonstrate the importance of this liver-to-brain axis in ameliorating age-related regenerative and cognitive impairments.^1^

**Fig. 1:**
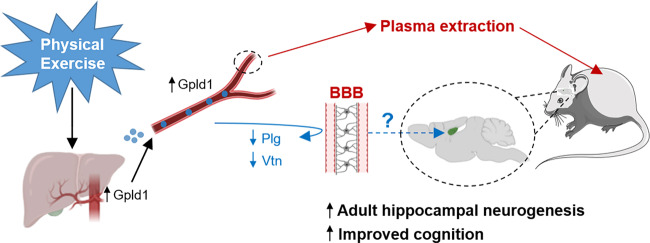
Model proposed by Horowitz et al.^5^ Physical exercise increases the expression of glycosylphosphatidylinositol (GPI)-specific phospholipase D1 (Gpld1) in the liver of aged mice, increases adult hippocampal neurogenesis (AHN) and improves cognition. The transfer of plasma from aged or mature exercised mice to aged sedentary counterparts mimics the beneficial effects of physical activity. The authors identify Gpld1 as a key molecule in mediating the effects of physical exercise on AHN and cognition in aged mice. BBB blood–brain barrier, Plg plasminogen, Vtn vitronectin

Physical activity exerts numerous beneficial actions on the brain and body. Exercise improves cardiovascular fitness and ameliorates metabolic syndrome, and other conditions related to a sedentary lifestyle.^2^ It contributes to preserving cognitive abilities in healthy adults and delays cognitive decline during physiological aging. Moreover, growing evidence points to neuroprotective effects of exercise in patients with mild cognitive impairment or even neurodegenerative conditions such as Alzheimer´s disease (AD). Nevertheless, although regular or high-intensity exercise is a widely available, inexpensive and affordable approach to maintain physical and psychological fitness for the general population, it may not be practicable for the elderly or during the course of distinct diseases. Consequently, remarkable efforts over recent decades have been made to identify molecules capable of partially or completely mimicking the positive effects of exercise on the brain. In this regard, physical exercise increases the levels of various circulating growth factors, such as brain-derived neurotrophic factor (BDNF), vascular endothelial growth factor (VEGF), insulin-like growth factor I (IGF-I), and irisin, among others. These observations led to the formulation of the *neurotrophic hypothesis* more than a decade ago, which proposes that exercise improves brain function by increasing the levels of circulating trophic factors.^2^ Although the precise mechanisms of action of these molecules remain to be fully elucidated, it is known that they are synthesized by a plethora of organs and tissues and that they exert their actions at multiple sites throughout the body. In particular, the liver–brain axis has a key role in transferring the effects of physical exercise to the brain, the hippocampus seemingly being one of the brain regions most intensely targeted by this stimulus (Fig. 1). This structure has key roles in memory, learning and mood regulation. Moreover, it hosts the generation of new neurons throughout life, a phenomenon named adult hippocampal neurogenesis (AHN). Among the multiple hippocampal targets of physical exercise, a sustained and robust increase in AHN rate is one of the most evident effects observed in the brains of exercised animals. The occurrence of AHN has been demonstrated across the entire mammalian phylogenetic scale, including rodents, humans and non-human primates.^3^ AHN confers an unparalleled degree of plasticity to the mammalian hippocampal circuitry and it is also believed to mediate some of the positive effects of physical exercise on cognition. Given that AHN is markedly impaired in patients with AD^4^ and animal models of neurodegenerative or psychiatric diseases, exercise emerges as a potentially interesting therapeutic strategy to prevent or slow down the progression of several pathological features of these diseases.

Previous work by Villeda et al.^5^ demonstrated that transferring blood from young to aged mice rejuvenates the systemic inflammatory milieu, increases AHN and improves cognition. The new study by Horowitz et al.^1^ takes one step further and shows that the beneficial effects of physical exercise on AHN and cognition can be transferred to sedentary aged mice by injecting them with the plasma of exercised animals (Fig. 1). They show that the benefits of receiving plasma injections from exercised mice occur regardless of the age (mature vs. aged) of the donor. Sedentary aged mice that received plasma from aged or mature exercised mice exhibited increased survival and differentiation rate of new neurons in the hippocampus, together with enhanced hippocampal-dependent learning. Importantly, the extent of these actions was similar to that observed after a period of voluntary wheel running in mice of the same age. Together with these inspiring results, the authors used isobaric tagging together with liquid chromatography–tandem mass spectrometry and found that the abundance of 12 circulating factors was increased in aged and young mice in response to physical exercise. Functional enrichment analysis of these factors identified a soluble factor synthesized in the liver as a critical mediator of the positive effects of physical exercise on the brain. The circulating levels of this molecule, named glycosylphosphatidylinositol (GPI)–specific phospholipase D1 (Gpld1), are increased after physical exercise both in mice and humans. Liver overexpression of Gpld1 in aged mice fully mimics the beneficial actions of physical exercise or plasma transfer on AHN and cognition. Moreover, to test whether the catalytic activity of systemic Gpld1 directly mediates its effects on adult neurogenesis and cognition, the authors generated expression constructs encoding Gpld1 with site-directed His-Asn mutations, which abolished its GPI-anchored substrate cleavage catalytic activity. They show that full integrity of the catalytic activity of Gpld1, which activates downstream pathways related to coagulation cascades, is required to achieve the aforementioned effects.^1^

The data obtained by Horowitz et al. bring to light the intricate complexity of the brain–body axis and how physical exercise reshapes several aspects of this (bi)directional communication. The authors generated expression constructs encoding a high-affinity nanoluciferase binary technology (HiBiT)-tagged version of Gpld1 to demonstrate that this molecule appears not to readily cross the blood–brain barrier (BBB) and enter the brain. Future research should seek to elucidate the precise mechanisms through which it elicits neuroprotective actions on AHN and cognition. Nevertheless, these data pave the way for the design of strategies aimed to transfer the beneficial effects of exercise to sedentary or diseased subjects. In this regard, alterations in AHN are observed even before the occurrence of massive neurodegeneration in the brains of AD patients,^4^ and these alterations have been proposed to be related to the impairments of episodic memory observed in these patients. Therefore, preventing the decline of AHN may modulate certain aspects of the pace of neurodegeneration in AD and other neurodegenerative or neuropsychiatric disorders. Given that the ability or willingness to regularly perform physical exercise may be greatly influenced by the mental or physical condition of each individual, the results obtained by Horowitz et al. may open up opportunities for the design of novel strategies to prevent or slow down the progression of neurodegenerative or psychiatric diseases or even reverse age-related cognitive impairments.
